# Analysis of role-play in medical communication training using a theatrical device the fourth wall

**DOI:** 10.1186/1472-6920-6-51

**Published:** 2006-10-14

**Authors:** Torild Jacobsen, Anders Baerheim, Margret Rose Lepp, Edvin Schei

**Affiliations:** 1 Section for General Practice, Department of Public Health and Primary Health Care, University of Bergen, Kalfarveien 31, N-5009 Bergen, Norway; 2 University College of Borås, Borås, Sweden

## Abstract

**Background:**

Communication training is a central part of medical education. The aim of this article is to explore the positions and didactic functions of the fourth wall in medical communication training, using a role-play model basically similar to a theatrical performance.

**Method:**

The empirical data stem from a communication training model demonstrated at an international workshop for medical teachers and course organizers. The model involves an actress playing a patient, students alternating in the role of the doctor, and a teacher who moderates. The workshop was videotaped and analyzed qualitatively.

**Results:**

The analysis of the empirical material revealed three main locations of the fourth wall as it moved and changed qualities during the learning session: 1) A traditional theatre location, where the wall was transparent for the audience, but opaque for the participants in the fiction. 2) A "timeout/reflection" location, where the wall was doubly opaque, for the patient on the one side and the moderator, the doctor and the audience on the other side and 3) an "interviewing the character" location where the wall enclosed everybody in the room. All three locations may contribute to the learning process.

**Conclusion:**

The theatrical concept 'the fourth wall' may present an additional tool for new understanding of fiction based communication training. Increased understanding of such an activity may help medical teachers/course organizers in planning and evaluating communication training courses.

## Background

Art is increasingly used in educational settings around the world especially when development of complex communicative skills and abilities is targeted. In health education there has been a growing awareness of the value of the use of actors and educational drama [1].

Standardised patients, both professional trained actors and ordinary people who has received training to present an illness in a standardised manner, have become commonplace in medical education. The most commonly used role-play format in medical communication training is roughly based on the three following points; a) Students interacting with each other, or with a standardised patient in a one to one situation, b) A standardised patient or an actor/actress running a complete consultation as a linear narrative, c) Students are given feedback or evaluated after the role-play.

This study is based on a specific role-play format we have developed for our medical consultation training. Our training model differs from all of the points above; a) The students interact with an actress acting the patient, b) The consultation is run as an interrupted narrative and c) Instead of using a feedback pedagogy, focusing on evaluation, the moderator together with the students reflect during timeouts on different possible ways to proceed, given the actual stage of the consultation.

A consequence of using actors and role-play in medical education is that a theatrical situation, a fiction, is created in the classroom. Manipulated time and space are the main characteristics of a fiction, and a theatrical situation is one where "A impersonates B while C looks on" [2]. *The fourth wall *is a metaphor designating "the space separating the audience from the action of a theatrical performance, traditionally conceived of as an imaginary wall completing the enclosure of the stage" [3].

When using fiction with actors and role-playing in medical teaching, the setting is similar to the theatre; some people are participating in a fiction, and some people are watching those who participate.

The aim of this article is to explore the positions and didactic functions of the fourth wall in medical communication training, using a role-play model basically similar to a theatrical performance.

### The fourth wall

The principle of The fourth wall is one of the best established conventions from the naturalistic period (about 1870–1900) in the history of dramatic art. It is one of the basic principles concerning the boundary within *communication *between the audience and the acting characters. The fourth wall is described literarily for the first time by the French dramatist and writer Jean Jullien in the essay collection *Le Théatre vivant *[4].

Si le comédien doit toujours suivre les impressions de la salle du bout de l'oreille, il doit ne rien en laisser paraître, jouer comme s'il était chez lui, sans se préoccuper de l'émotion qu'il soulève, des bravos ou des chuts; il faut que l'emplacement du rideau soit un quatrième mur transparent pour le public, opaque pour le comédien [4].

This "mur", opaque for the actors is the naturalistic theatre's foundation. It was established to increase the illusion of *real life *on the stage. The naturalistic theatre's fourth wall made it possible for the actors to behave onstage as they would in a real room in real life, and the audience could observe the fiction "as if" they were observing real life [5]. The imaginary fourth wall between the audience and the acting characters enabled a more direct interacting between the characters with more natural vocal delivery and smaller gestures, in stead of large stylized acting. These changes were possible behind a fourth wall, "transparent for the audience, opaque for the actors".

The principle of the fourth wall is still alive, especially in traditional Western theatre. Throughout the history of the theatre the wall has been overseen, smashed down or expanded according to the effects intended on the communication between audience and actors [6].

Bertold Brecht (1898–1956) and Augusto Boal (1931 –) were both pioneering practitioners engaged in the theatrical communication process, and they both utilized the fourth wall didactically. Brecht's new artistic principles for the scientific age, *The Verfremdungseffekt *(A-effect), concerned different devices to make the spectator adopt an attitude of inquiry and critical reflection on a conscious plane about the incident, instead of an emotional identification with the characters on a subconscious plane [7]. By breaking the fourth wall and addressing the audience directly from the stage, he was one step closer to the 'de-involvement' he wanted to achieve:

The first condition for the achievement of the A-effect is that the actor must invest what he has to show with a definite gest of showing. It is of course necessary to drop the assumption that there is a fourth wall cutting the audience off from the stage and the consequent illusion that the stage action is taking place in reality and without an audience. That being so, it is possible for the actor in principle to address the audience direct [8].

Augusto Boal believed that Brecht's theatre was an improvement with respect to audience awareness, but Boal went one step further than Brecht, and encouraged the audience not only to think for themselves but also to act/take action, because Boal was of the opinion that only through action do we achieve true insight [9]. In Boal's *Forum Theatre *the audience is invited to oversee the fourth wall and participate in the fiction onstage to intervene and change the behavior of the oppressed protagonist, in order to find the most effective method of dealing with the oppression [10].

## Methods

In 2003 two of the authors, TJ and AB, developed an interactive, inter-disciplinary model for communication skills training in medical education at the University of Bergen, Norway [11,12]. This training model has since then been used as a part of a consultation course for final year medical students in Bergen. The course is obligatory and the students usually amount to a group of 30 participants.

### The training model

The training model consists basically of the following points:

1. The fiction: Beside the table at the front of the room sits an actress acting the patient and a medical student carrying out the consultation. The moderator is standing outside the fiction (see location 1). The other students make up the audience.

2. Timeout: The moderator decides to take frequent timeouts for discussing the next step in the consultation at critical incidents, that means: when the students obviously struggle or at apparent crossroads in the consultation. He also takes timeout when the consultation runs too smoothly.

3. Continuation of the consultation: After the timeout discussion, another student takes the doctor's seat, trying out his/her own options, or one of the options proposed by someone else in the audience.

This model is being utilized for training the students in three different themes which asks for different communicating strategies; 1) How to communicate with the shy and withdrawn patient 2) Breaking bad news and 3) How to communicate with the aggressive patient.

We use a professional actress to act the patient part. She needs to have competence in staging complex characters combined with the ability to adjust to the competence of the individual student.

This training model requires of the moderator that he/she has the ability to lead a group process and having relevant pedagogical and medical competence for the special communication skills relevant for the three themes.

Throughout the whole consultation, it is the moderator who starts the timeouts for reflection with the students. Our moderator may be regarded as a facilitator of the process and as a parallel to Boal's joker in Forum Theatre [9].

The joker, like our moderator, acts as an interpreter of a collective process, but different strategies are not discussed with Boal's joker. His Forum Theatre performances always demonstrate a mistake, a failure where the audience is invited to solve a problem by interacting directly in the performance and through this interaction, find new strategies to stand up against oppression [9].

Our training model focuses on possibilities for reflection on the ongoing process combined with the opportunity to try out one of the options being discussed, and the moderator underlines that there are no right standard answers. Everyone is encouraged to develop and try out their own consultation style based on their actual competence.

Our model's basic pedagogical strategy is the commuting between being *in *the fictive consultation and the discussions of what to do next, *outside *the fiction. Timeouts give the students opportunities to perceive, interpret and discuss the fiction together with the moderator and propose changes and strategies to be tried out as the consultation restarts after timeout.

The purpose of the training model is a pragmatic one; to give the individual student a group based opportunity to act, re-act and reflect on possible consultation strategies as the consultation evolves. By participating, the students might increase his/her preparedness for similar situations in real life.

### The empirical data and analysis

This training model was run similar to what we do for the students at an international workshop for medical teachers and course organizers in 2005. The workshop lasted for one hour and involved about 50 participants. The first author was performing the part of the patient and the second author was moderating the workshop.

The whole workshop was videotaped and transcribed word by word with time specifications for each line of speech and notes about the most striking nonverbal language.

During the transcription we discovered, however, that the fourth wall was first and foremost a *spatial *phenomenon which appeared only on the video recording and could not be found in the written transcription. For this reason, in order to be able to analyse the relationship between the fourth wall and the didactic room, we had to return to the video recording, since this was the only material which fully documented the changes in the room. The video was then watched again, this time in a *Close reading *[13] perspective with the general text concept widened to concern all aspects of the video as 'text'. Close reading is as a research method within literary studies to analyse the text's specific elements, but this analytic praxis also occurs in other disciplines (e.g. film studies). Close reading is used to produce evidence for theorization, focusing on the aesthetic object and not its effect.

In this study, Close reading was used to produce detailed information about the connection between the fourth wall, as a phenomenon, and the learning process. This Close reading step included the second author as a co-researcher.

Since the first author had a double role in the project, being a researcher and the performing actress, we wanted to obtain a certain distance during analysis. For this reason we chose to use video as a research tool. This was also one reason why two researchers studied the video.

Ethical approval: Not applicable.

## Results

The aim of this study was to explore where and how the fourth wall exists in a communication training model. The empirical material revealed three different main locations of the fourth wall:

### **Location 1*** – A traditional theatre location*

**Figure 1:**
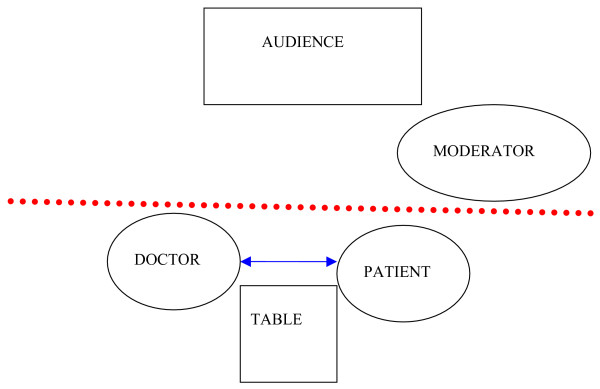
Location 1. The arrows indicate the direction of the communication. The fourth wall is transparent for the audience, opaque for the actors.

From the moment the actress introduced herself in role as a patient and the moderator invited a participant to be the doctor to interact with the patient, the consultation began and this situation may be described as a traditional theatre situation where "A impersonates B while C looks on", and the fourth wall acted as "un quatrième mur transparent pour le public, opaque pour le comédien" [4], i.e. the fourth wall was positioned between those acting and those watching (Figure 1).

### **Location 2*** – A timeout/reflection location*

**Figure 2:**
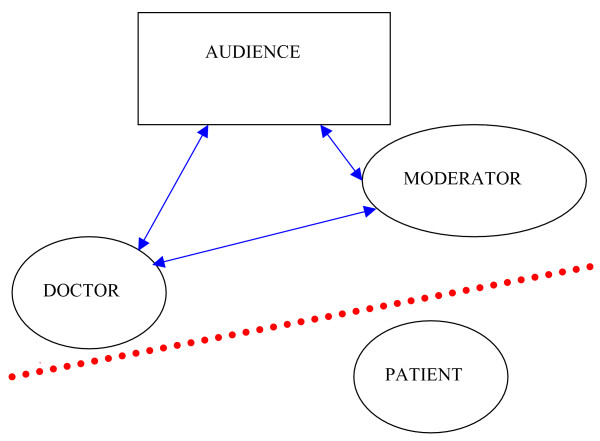
Location 2. The fourth wall enables a discussion about the patient between the doctor, the audience and the moderator.

At the first critical incident of the consultation between the doctor and the patient/actress, the moderator stopped the ongoing dialogue by taking a timeout. The doctor stopped the interaction with the patient and the patient minimized her response to the situation (not by going into a "freeze position" but by taking a general non-responsive position). The moderator addressed both the doctor and the audience and asked for possible options onwards. When moderator took timeout, the wall moved from the traditional theatre position, where the wall was transparent for the audience, to a totally new position where the wall functioned as a soundproof and opaque wall between the patient/actress on the one side and all the others in the room on the other side (Figure 2). In that situation the doctor, the audience and the moderator could discuss the patient *as if she were not present*. One might even say that the wall, in this situation, was doubly opaque because the patient was non-responsive to the options being discussed and because the moderator communicated with everyone in the room except the patient.

### **Location 3*** – An interviewing the character location*

**Figure 3:**
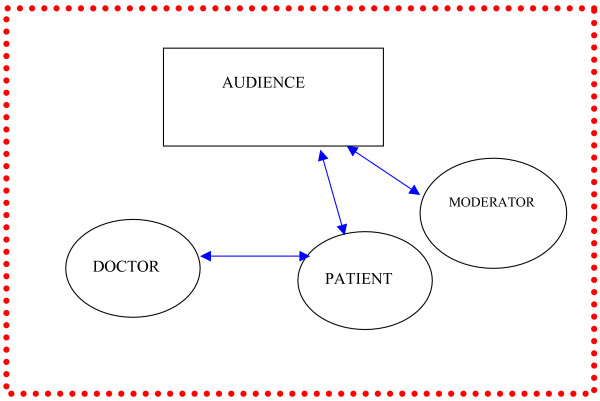
Location 3. The remove of the fourth wall opens up for a possible communication between everybody in the room.

At some point in the process of the consultation the moderator stopped the ongoing fiction, not by taking timeout, but by informing the audience that everyone had the possibility to pose questions directly to the patient from their places. Hence everybody in the room were verbally and emotionally involved in the fictional context. The framing was 'a doctor's office' and the audience was a kind of multi-headed doctor. There was no wall separating fiction and reality because the fictional and the real contexts had melted into one, and the wall was standing as "un mur" enclosing the fiction in the classroom (Figure 3).

## Discussion

We found three main locations of the fourth wall, all of which may have consequences for the learning process: 1) A traditional theatre location, where the wall was transparent for the audience, opaque the other way. 2) A timeout/reflection location where the wall was doubly opaque and isolated the patient from everybody else in the room and 3) an interviewing of the character location where the wall expanded and enclosed everybody in the room.

In the following discussion, we will elucidate these consequences in the light of the theories of Bertold Brecht and Augusto Boal. We will also investigate a part of the training model's connection to drama pedagogy with the drama convention 'Teacher-in-role' [19] as a starting point.

**Location 1**, the traditional theatre location, is the only part of the training model identical to the naturalistic theatre's use of the fourth wall as described by Jullien. We have found, in a previous study, that the audience experienced this location as being both emotionally and intellectually stimulating [12]. The audience reported that *watching *the interaction between the patient and the doctor triggered reflection strongly and on a par with actively performing the doctor's role. The combination of reflection and action in our model is inline with drama theory focusing on "learning by doing and reflecting" [14], emphasizing learning through practical action and experiences and not just through witnessing the action. Schön describes how professional competence is linked to an ability to try out ideas on-line and to understand how these trials might lead to improvement within the workplace context [15,16].

*If *the patient or the doctor had broken the opaque wall facing the audience in this first location, the fiction's credibility would have been weakened and accordingly the participants' learning outcome would have been reduced.

The interacting doctor is in a *dual perception *position [17] because of the muting between being outside the fiction, in the timeouts, and inside the fiction when interacting with the patient/actress. He/she is moving to and fro between the experiences of "I am making it happen" and "it is happening to me" [18]. This unique position of being emotionally and intellectually involved at the same time is regarded as one of the most potent learning positions in drama pedagogy [10].

**Location 2**, the 'timeout/reflection' location, is more complex than location 1. The very moment the moderator took timeout, communication patterns changed. The empirical material revealed that the *absence of communication *between the doctor and the patient in location 2, combined with the new direction of communication between the moderator and the on-lookers, made the wall doubly opaque. The patient/actress contributed to this through a passive, withdrawn and non-responsive body language. The 'doubly opaque-situation' made it possible for the participants and the moderator to discuss the patient *as if she were not there *even though she was sitting among them. To discuss a patient as the consultation evolves is clearly unethical in real life because of the need to protect the dignity of the patient. An actress will be less vulnerable because of her professional handling of the role. The students are reminded of the obvious that in real life patients should never be discussed without taking notice of the patient's presence.

The presence-absence of the patient/actress may stimulate the audience's reflective thinking about the challenges of the portrayed medical situation. As the audience was invited to suggest the next steps in the consultation, the fourth wall was disrupted in a Brechtian way, and through these verbalized reflections, heard by both subsequent "doctors" and by the patient/actress, the participants became co-writers of the further development of the fiction.

In **location 3**, the audience was invited to ask questions directly to the patient from their seats. Because the patient in our context does not have a fixed script, she can be further invented, reinvented and rearranged within the situation, through the actresses' improvisations. The audience was instructed not to ask evaluating questions concerning the doctor's performance in the consultation process, but was invited to ask questions concerning the patient's concrete experiences in the interaction. This method stems from the common drama technique *teacher-in-role *developed in England by Dorothy Heathcote in the seventies. This convention is often utilized in Theatre in Education [19] in various ways for exploring a character or investigating the situation as perceived by the character [20].

In our context, the moderator invited the audience into a dialogue with the patient through breaking the fourth wall, and the patient contributed by making eye contact with the audience and by responding to all questions. Again, we are quite close to Brecht's didactical method, but one might say that we bring the audience one step closer to the fiction then Brecht did by allowing them to interact verbally.

This active position for the audience is as close to a real consultation situation as possible. The purpose is to create a safe "room" where the participants may develop and test their own curiosity and ideas concerning the patient, her needs, her experience of the situation, and what might be helpful inn a challenging consultation.

Augusto Boal believed Brecht's theatre to be an improvement upon the Aristotelian theatre, since the viewer is encouraged to think independently. However, Boal proposed that this was insufficient; the viewer must take action in order for change to take place, and only *through our own actions *do we achieve true insight [9]. This thinking is manifested first and foremost in Boal's Forum Theatre where the viewer goes from being passive to breaking through the fourth wall and becoming an active, *action-taking *participant. Boal's didactical method may, in principle, resemble our training model; we have in common the aim of wishing to stimulate to individual reflection through action and be a bit more prepared to meet the unknown through experimental learning.

### Strengths and limitations of the study

The most important thing to point out here is that the transcription proved to be a dead end in this project. It was a challenge to accept this because the transcription process had been particularly time consuming. However, when we discovered that the transcription could only tell us about interpretations of the verbal material in the room, and not about the metaphorical spatial concept, it became clear that we had to go back to the video recordings in our further analysis.

The first author's dual position, as an actress performing the role of the patient and as a researcher, might have been a limitation. However, we worked out some strategies to reduce this problem. This is being described in the Method section.

## Conclusion

The theatrical concept 'The fourth wall' may represent an additional tool for new understanding of fiction based communication training. Increased understanding of such a complex activity may help medical teachers/course organizers in planning and evaluating communication training courses.

## Competing interests

The author(s) declare that they have no competing interests.

## Authors' contributions

TJ conceived of the study, decided on design, provided the empirical material, carried out the analysis and drafted the manuscript. AB was a co-researcher and participated at all the stages of the study. ES and ML contributed to the research questions, decision on design, and data analysis. All authors read and approved the final manuscript.

## Pre-publication history

The pre-publication history for this paper can be accessed here:
